# Analytical validation of a high-resolution melting assay for UGT1A1 TATA-box polymorphisms

**DOI:** 10.1007/s11033-026-12240-9

**Published:** 2026-07-08

**Authors:** Stefano Gambarino, Anna Clemente, Anna Pau, Cristina Calvi, Paola Montanari, Ilaria Galliano, Massimiliano Bergallo

**Affiliations:** 1 Present Address: Department of Public Health and Pediatric Sciences, Immunopathology Laboratory, Medical School, University of Turin, Turin, 10126 Italy; 2 BioMole srl, Via Petrarca 4/F, 10126 Turin, Italy; 3 Laboratory of Specialistic Pediatry, Department of Children’s Pathology and Care, Regina Margherita Children’s Hospital, Piazza Polonia 94, Turin, 10126 Italy

**Keywords:** UGT1A1, High-resolution melting, Real-time PCR, Pharmacogenetics, Analytical validation

## Abstract

**Background:**

Polymorphisms in the TATA-box of the UGT1A1 promoter are responsible for Gilbert syndrome and play a key role in irinotecan-related toxicity. Reliable, rapid, and cost-effective genotyping methods are therefore required in routine molecular diagnostics.

**Methods and Results:**

We performed an analytical validation of a High-Resolution Melting (HRM) assay designed to discriminate UGT1A1 TATA-box alleles. A total of 106 neonatal clinical samples were analyzed to assess genotype distribution under routine diagnostic conditions. Analytical validation was performed using plasmid controls, reference genomic DNA samples, and sequencing-confirmed in-house samples. Plasmid-derived TA5, TA6, TA7, and TA8 genotypes and their heterozygous combinations were tested in quintuplicate. Reference genomic DNA samples carrying rare or non-observed genotypes were tested in triplicate, and 30 in-house samples representative of TA6/TA6, TA6/TA7, and TA7/TA7 genotypes were tested in triplicate and confirmed by Sanger sequencing. The HRM assay discriminated the tested genotypes based on melting temperature (Tm) and curve morphology. HRM genotype calls showed complete concordance with expected or reference genotypes. Intra-test analysis of the TA6/TA7 standard showed a mean Tm of 43.36 °C, SD of 0.20 °C, and CV of 0.46%, while inter-test analysis showed identical Tm values across replicates.

**Conclusions:**

The UGT1A1 TATA-box HRM assay showed reproducible genotype discrimination in the tested validation panel and may represent a practical approach for routine UGT1A1 promoter genotyping.

## Introduction

The UGT1A1 gene encodes uridine diphosphate glucuronosyltransferase 1A1 (UGT1A1), a key phase II metabolic enzyme responsible for the glucuronidation of bilirubin and a wide range of endogenous and exogenous compounds, including steroid hormones and several clinically relevant drugs [1, 2]. Variations in UGT1A1 expression or activity may therefore result in both benign and clinically significant phenotypes.

One of the most extensively studied regulatory regions of UGT1A1 is the TATA-box located in the promoter region. This element contains a polymorphic dinucleotide repeat (TA)n, where the number of TA repeats modulates transcriptional efficiency. The wild-type allele (UGT1A1*1) is characterized by six TA repeats (A[TA]6TAA), whereas the most common variant allele, UGT1A1*28, contains seven repeats (A[TA]7TAA) and is associated with reduced transcriptional activity [3]. Less frequent alleles, such as UGT1A1*36 (five repeats) and UGT1A1*37 (eight repeats), have also been described and are associated with increased or further reduced promoter activity, respectively [3].

The clinical relevance of these polymorphisms is well established. Homozygosity for UGT1A1*28 represents the molecular basis of Gilbert syndrome, a benign condition characterized by chronic, mild unconjugated hyperbilirubinemia, often detected during childhood or adolescence [2]. In neonatal and pediatric settings, persistently elevated unconjugated bilirubin levels in the absence of hemolysis or liver disease may prompt molecular testing to support differential diagnosis and clinical management.

Beyond bilirubin metabolism, UGT1A1 polymorphisms play a crucial role in pharmacogenetics. Reduced UGT1A1 activity has been associated with increased toxicity of irinotecan, a chemotherapeutic agent widely used in colorectal cancer, due to impaired glucuronidation of its active metabolite SN-38 [4, 5]. As a consequence, several international guidelines recommend UGT1A1 genotyping prior to irinotecan administration in selected clinical contexts [6].

From a laboratory perspective, accurate and rapid determination of UGT1A1 TATA-box polymorphisms is therefore essential. While Sanger sequencing is generally considered the reference method for sequence analysis, it is relatively time-consuming and costly for routine diagnostic workflows. Alternative approaches, including allele-specific real-time PCR assays, have been proposed for targeted detection of UGT1A1 variants [7], although these strategies may be limited when multiple alleles must be discriminated simultaneously. High-Resolution Melting (HRM) analysis represents an attractive alternative, allowing post-PCR discrimination of sequence variants based on their melting behavior and curve morphology [8, 9].

The present study reports an analytical validation of a commercial HRM assay designed for the detection of UGT1A1 TATA-box polymorphisms. The validation was conducted on a cohort of clinical samples routinely referred for UGT1A1 testing, with the aim of assessing genotype discrimination capability, reproducibility, and overall robustness of the method.

## Materials and methods

### Samples and clinical context

A total of 106 neonatal clinical samples were included to evaluate genotype distribution and assay performance in routine diagnostic conditions. The cohort included 63 males (59.4%) and 43 females (40.6%), of whom 90 were term neonates (> 37 weeks of gestation; mean birth weight 3250 ± 500 g) and 16 were preterm neonates (< 37 weeks of gestation; mean birth weight 2200 ± 750 g). Genomic DNA samples extracted from EDTA-anticoagulated whole blood were analyzed. Samples were collected during routine clinical activity. In a substantial proportion of cases, the test request originated from neonatal units (Regina Margherita Children’s Hospital) due to the presence of persistent unconjugated hyperbilirubinemia.

Based on available clinical information, mean total serum bilirubin levels at the time of test request ranged between 2.5 and 4.0 mg/dL, with unconjugated bilirubin predominance and absence of hemolytic disease or structural liver abnormalities. These biochemical findings were considered suggestive of an inherited alteration of bilirubin metabolism and justified molecular investigation of the UGT1A1 promoter region.

### DNA extraction and quantification

Genomic DNA was extracted from whole blood samples using automated extraction systems (Maxwell^®^ MDx or Maxwell^®^ RSC, Promega) with validated blood DNA extraction kits, according to the manufacturer’s instructions. DNA concentration and purity were assessed spectrophotometrically, and samples were diluted to a working concentration of 10 ng/µL prior to amplification.

### HRM assay and instrumentation

Genotyping was performed using the UGT1A1 TATA-Box HRM kit (BM-044) (BioMole, Turin, Italy), which includes a ready-to-use master mix and an internal positive control corresponding to the UGT1A1*1/*28 genotype. High-Resolution Melting (HRM) analysis was performed using a CFX96 Real-Time PCR Detection System (Bio-Rad Laboratories, Hercules, CA, USA), in accordance with the manufacturer’s technical recommendations for HRM assays. Instrument calibration, optical acquisition settings, and data analysis parameters were set following Bio-Rad guidelines for HRM analysis to ensure optimal temperature resolution and reproducibility.

The thermal profile consisted of polymerase activation at 95 °C for 2 min, followed by 55 amplification cycles (95 °C for 15 s, 60 °C for 30 s, 72 °C for 30 s). High-resolution melting analysis was performed from 35 °C to 60 °C with temperature increments of 0.4 °C.

### Analytical validation design

Analytical validation was performed using synthetic plasmid controls, reference genomic DNA samples, and sequencing-confirmed in-house genomic DNA samples. Plasmids corresponding to the TA5, TA6, TA7, and TA8 UGT1A1 TATA-box sequences were purchased from Eurofins. After reconstitution, plasmids were tested individually or in defined combinations to generate a validation panel including TA5, TA5/6, TA5/7, TA5/8, TA6, TA6/7, TA6/8, TA7, TA7/8, and TA8 genotypes. Each plasmid-derived genotype was tested in quintuplicate.

Reference genomic DNA samples carrying rare or non-observed genotypes in the clinical cohort were obtained from the Coriell Institute. These included TA5/6, TA5/7, TA5/8, TA6/8, and TA7/8 genotypes and were tested in triplicate. In addition, 30 in-house genomic DNA samples representative of the genotypes observed in the clinical cohort, including 10 TA6, 10 TA6/7, and 10 TA7 samples, were tested in triplicate and confirmed by Sanger sequencing of the UGT1A1 promoter region encompassing the TATA-box. These 30 samples were selected to represent the three genotype categories observed in the clinical cohort, with 10 samples per genotype category. Samples were selected to cover the observed HRM genotype clusters.

Intra-test and inter-test reproducibility were assessed using the TA6/7 standard. Five replicates were analyzed within the same analytical session to assess intra-test repeatability, and five replicates were analyzed across independent analytical sessions to assess inter-test reproducibility. Repeatability and reproducibility analyses were performed under routine laboratory conditions by different experienced laboratory personnel. However, operator-to-operator variability was not designed as a separate formal validation endpoint. Genotype assignment was evaluated by considering melting temperature values, normalized melting curves, difference plots, and clustering behavior.

### Controls and quality criteria

Each analytical run included a no-template control (NTC) to monitor contamination and a positive control/standard to verify amplification efficiency and melting temperature accuracy. Runs were considered valid only when the NTC showed no amplification signal and the positive control/standard generated the expected melting profile.

Genotype calls were considered acceptable when replicate samples showed concordant genotype assignment and when melting profiles clustered consistently according to Tm values, normalized melting curves, and difference plots. Because plasmid-derived controls may show slightly different absolute Tm values compared with genomic DNA samples, genotype assignment was not based on fixed Tm thresholds alone.

### Data analysis

Melting curves were analyzed using both the instrument software and Precision Melt Analysis™ Software. Genotype assignment was based on the combined evaluation of Tm values, normalized melting curves, difference plots and clustering behavior. Analytical performance was assessed in terms of genotype discrimination, replicate concordance, concordance with expected or reference genotypes, and intra-test/inter-test reproducibility.

For reproducibility assessment, mean Tm, standard deviation, coefficient of variation, and Tm range were calculated. Hardy–Weinberg equilibrium (HWE) was evaluated as a descriptive population-level analysis of the observed clinical cohort. Allele frequencies were calculated by gene counting, and expected genotype frequencies were derived accordingly. Deviation from HWE was tested using the chi-square (χ²) goodness-of-fit test and an exact test, with *p* < 0.05 considered statistically significant.

## Results and Discussion

### Genotype distribution

All 106 samples yielded interpretable HRM profiles, and no failed or marginal melting curves were observed among the samples included in the study.

The observed genotype distribution is summarized in Table 1.

**Table 1:** Distribution of UGT1A1 TATA-box genotypes in the clinical cohort

Genotype	TA repeats	*n*	%
UGT1A1*1/*1	6/6	42	39.6
UGT1A1*1/*28	6/7	45	42.5
UGT1A1*28/*28	7/7	19	17.9

*n =* number of samples

Each genotype observed in the clinical cohort was associated with a characteristic melting profile, allowing clear discrimination between homozygous and heterozygous states.

The distribution of UGT1A1 TATA-box genotypes was consistent with Hardy–Weinberg equilibrium as a descriptive population-level observation. Based on the observed allele frequencies, the expected numbers of homozygous and heterozygous genotypes did not differ significantly from those observed (χ² = 1.26, *p* = 0.262). This finding was confirmed by an exact test for Hardy–Weinberg equilibrium (*p* = 0.307).

### HRM curve analysis

**Fig. 1:**
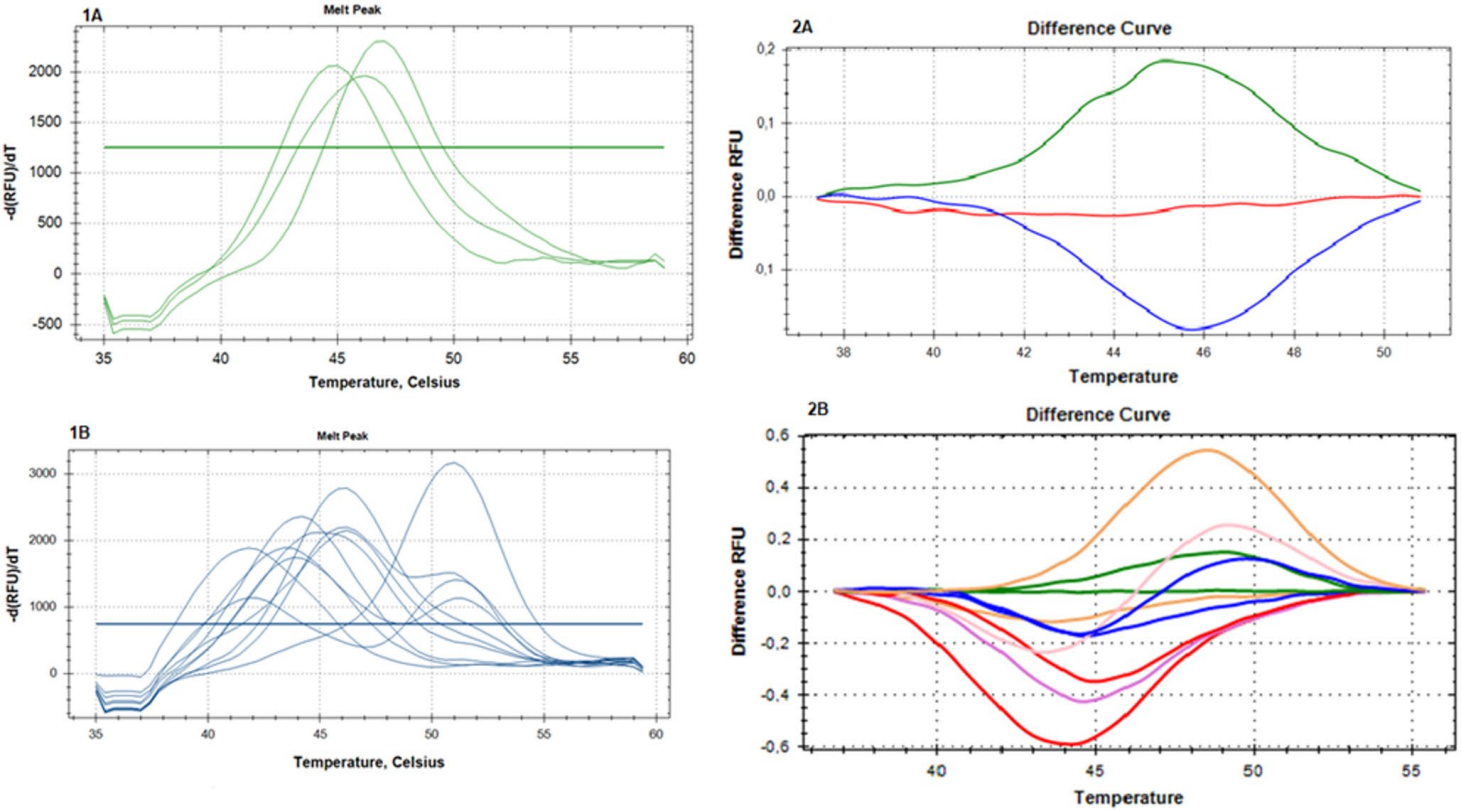
Representative HRM profiles of UGT1A1 TATA-box genotypes 1–2 **A**: Melt peak profiles and difference curves from clinical genomic DNA samples representative of the genotypes observed in the cohort: TA6/TA6, TA6/TA7, and TA7/TA7. **1B**–**2B**: Melt peak profiles and difference curves from the analytical validation panel, including TA5–TA8 alleles and heterozygous combinations. Genotype calls were assigned by combining melting temperature values, curve morphology, difference plots, and clustering behavior

Representative HRM profiles are shown in Fig. 1. Melt peak profiles and difference curves obtained from clinical genomic DNA samples allowed discrimination of the genotypes observed in the cohort, namely TA6/TA6, TA6/TA7, and TA7/TA7. Additional HRM profiles obtained from the analytical validation panel supported discrimination of TA5–TA8 alleles and heterozygous combinations. Genotype assignment was based on the combined evaluation of melting temperature, curve morphology, difference plots, and clustering behavior.

### Analytical validation and reproducibility

Analytical validation was performed using plasmid controls, reference genomic DNA samples, and sequencing-confirmed in-house genomic DNA samples, as summarized in Table 2.

**Table 2:** Analytical validation panel of the UGT1A1 TATA-box HRM assay

Material	Genotypes evaluated	Replicates	Reference / expected genotype	Outcome
Eurofins plasmid controls	TA5, TA5/6, TA5/7, TA5/8, TA6, TA6/7, TA6/8, TA7, TA7/8, TA8	5 per genotype	Synthetic plasmid sequence / defined plasmid combinations	Concordant genotype discrimination
Coriell reference genomic DNA	TA5/6, TA5/7, TA5/8, TA6/8, TA7/8	3 per sample	Coriell reference genotype	100% concordance
In-house genomic DNA	TA6, TA6/7, TA7	10 samples per genotype, tested in triplicate	Sanger sequencing	100% concordance

The plasmid validation panel included TA5–TA8 alleles and their heterozygous combinations. Each plasmid-derived genotype was tested in quintuplicate and showed reproducible melting profiles, allowing discrimination of all tested genotypes. Reference genomic DNA samples carrying TA5/6, TA5/7, TA5/8, TA6/8, and TA7/8 genotypes showed concordant genotype assignment across triplicate analyses. The 30 in-house genomic DNA samples representative of the genotypes observed in the clinical cohort were also tested in triplicate and confirmed by Sanger sequencing, showing complete concordance with HRM genotype calls.

Intra-test and inter-test reproducibility were evaluated using the TA6/7 standard, as shown in Table 3.

**Table 3:** Intra-test and inter-test reproducibility of the TA6/7 standard

Assessment	Replicates	Tm range, °C	Mean Tm, °C	SD, °C	CV, %
Intra-test repeatability	5	43.2–43.6	43.36	0.20	0.46
Inter-test reproducibility	5	43.2–43.2	43.20	0.00	0.00

SD: standard deviation; CV: coefficient of variation; Tm: melting temperature

Intra-test analysis showed a Tm range of 43.2–43.6 °C, with a mean Tm of 43.36 °C, standard deviation of 0.20 °C, and coefficient of variation of 0.46%. Inter-test analysis showed identical Tm values of 43.2 °C across replicates.

Overall, all tested replicates produced concordant genotype calls. Genotype discrimination was supported by the combined evaluation of Tm values, normalized melting curves, difference plots, and clustering behavior. No amplification was observed in no-template controls.

### Discussion

This study reports the analytical validation of an HRM assay for UGT1A1 TATA-box genotyping in a routine diagnostic setting. The main contribution of the study is not the identification of novel UGT1A1 promoter alleles, which are already well established, but the structured validation of a closed-tube HRM workflow applicable to routine molecular diagnostics. In particular, the validation strategy combined clinical samples, plasmid-derived controls, reference genomic DNA samples, and sequencing-confirmed in-house samples, allowing assessment of genotype discrimination across the TA5–TA8 allelic range.

HRM analysis provides genotype discrimination by combining target amplification with post-PCR evaluation of melting behavior. This approach has been widely used for variant scanning and genotyping because sequence differences can alter melting profiles and curve morphology [8–10]. In the present assay, genotype assignment was based on the combined assessment of Tm values, melt peak profiles, normalized/difference curves, and clustering behavior. This combined interpretation is particularly relevant for TA-repeat polymorphisms, for which heterozygous samples may generate complex curve shapes related to heteroduplex formation [11]. The closed-tube format also reduces post-PCR handling and supports implementation in laboratories already equipped with HRM-capable real-time PCR platforms [12].

In the clinical cohort, only TA6/TA6, TA6/TA7, and TA7/TA7 genotypes were observed. However, TA5 and TA8 alleles were specifically included in the analytical validation using plasmid-derived controls and reference genomic DNA samples carrying rare or non-observed genotypes. Therefore, the clinical cohort and the analytical validation panel should be considered complementary: the former reflects routine diagnostic findings in the tested population, whereas the latter supports genotype discrimination across the TA5–TA8 allelic range.

Hardy–Weinberg equilibrium analysis was retained as a descriptive population-level assessment of the clinical cohort. However, it was not considered a primary measure of analytical accuracy, particularly because the cohort consisted of neonatal samples referred for diagnostic testing rather than a population-based sample. Analytical performance was primarily supported by replicate concordance, correct classification of plasmid and reference genomic DNA materials, and concordance with Sanger sequencing in the tested in-house samples. The assay may be useful in routine settings where rapid UGT1A1 promoter genotyping is required, including diagnostic evaluation of unconjugated hyperbilirubinemia and pharmacogenetic screening. UGT1A1 promoter variants are clinically relevant both in Gilbert syndrome and in irinotecan toxicity risk assessment [2, 4–6]. Nevertheless, some limitations should be acknowledged. The validation was performed on a single real-time PCR platform, and the rarest genotypes were assessed mainly using plasmid controls and reference genomic DNA rather than large numbers of routine clinical samples. Although repeatability and reproducibility analyses were performed by different experienced laboratory personnel, a dedicated operator-to-operator variability study was not included as a separate validation endpoint. In addition, the assay was evaluated using DNA samples adjusted to the working concentration specified in the protocol, and systematic robustness testing across broader DNA concentration ranges or variable DNA quality conditions was not performed. The clinical information reported in this study was used only to describe the diagnostic context in which UGT1A1 testing was requested. The study was not designed to assess clinical outcomes or genotype–phenotype correlations, and no formal analysis of the relationship between UGT1A1 genotype and bilirubin levels was performed. Therefore, clinical implications should be interpreted cautiously, and the results should be considered as supporting analytical performance rather than clinical efficacy. Future multicenter studies including larger and more diverse cohorts would be useful to further assess assay transferability, clinical utility and genotype–phenotype correlations.

## Conclusions

The UGT1A1 TATA-box HRM assay showed reproducible genotype discrimination across the tested validation panel, including plasmid controls, reference genomic DNA samples, and sequencing-confirmed in-house samples. Its closed-tube format may support implementation in routine molecular diagnostics, although further multicenter studies are needed to confirm transferability across platforms and clinical settings and to assess clinical utility and genotype–phenotype correlations.

## Data Availability

Not applicable.
